# Ultrastaging of lymph node in uterine cancers

**DOI:** 10.1186/1756-9966-29-5

**Published:** 2010-01-21

**Authors:** Corinne Bézu, Charles Coutant, Marcos Ballester, Jean-Guillaume Feron, Roman Rouzier, Serge Uzan, Emile Daraï

**Affiliations:** 1 Department of Gynaecology and Obstetrics, Hôpital Tenon, Assistance Publique des Hôpitaux de Paris, CancerEst, Université Pierre et Marie Curie, Paris 6, France

## Abstract

**Background:**

Lymph node status is an important prognostic factor and a criterion for adjuvant therapy in uterine cancers. While detection of micrometastases by ultrastaging techniques is correlated to prognosis in several other cancers, this remains a matter of debate for uterine cancers. The objective of this review on sentinel nodes (SN) in uterine cancers was to determine the contribution of ultrastaging to detect micrometastases.

**Methods:**

Review of the English literature on SN procedure in cervical and endometrial cancers and histological techniques including hematoxylin and eosin (H&E) staining, serial sectioning, immunohistochemistry (IHC) and molecular techniques to detect micrometastases.

**Results:**

In both cervical and endometrial cancers, H&E and IHC appeared insufficient to detect micrometastases. In cervical cancer, using H&E, serial sectioning and IHC, the rate of macrometastases varied between 7.1% and 36.3% with a mean value of 25.8%. The percentage of women with micrometastases ranged from 0% and 47.4% with a mean value of 28.3%. In endometrial cancer, the rate of macrometastases varied from 0% to 22%. Using H&E, serial sectioning and IHC, the rate of micrometastases varied from 0% to 15% with a mean value of 5.8%. In both cervical and endometrial cancers, data on the contribution of molecular techniques to detect micrometastases are insufficient to clarify their role in SN ultrastaging.

**Conclusion:**

In uterine cancers, H&E, serial sectioning and IHC appears the best histological combined technique to detect micrometastases. Although accumulating data have proved the relation between the risk of recurrence and the presence of micrometastases, their clinical implications on indications for adjuvant therapy has to be clarified.

## Introduction

Studies conducted in the 90's reported that sensitive immunochemical and/or molecular techniques used to analyze lymph nodes likely to contain metastases could assess the clinical stage to a previously unachievable degree of accuracy (ultrastaging) [1,2]. In a 1997 study of 595 patients with melanoma, Joseph et al evaluated the contribution of serial sectioning, immunohistochemistry (IHC) and a molecular technique with reverse transcriptase polymerase chain reaction (RT-PCR) to routine hematoxylin and eosin (H&E) histology to detect lymph node metastases. The study showed that routine H&E histology identified 73.8% of all metastases [3]. The remainder was detected by serial sectioning (7.8%) and IHC staining (18.4%) [3]. Moreover, RT-PCR upstaged 47% of the negative sentinel lymph nodes (SLN) [3]. In breast cancer, Cote et al reported that serial sectioning and IHC were able to detect respectively 7% and 20% of metastases in negative lymph nodes on H&E histology [1]. In 2001, a multicenter study of stage I-III colorectal cancer by Saha et al. reported that serial sectioning and IHC detected lymph node micrometastases in 14% of patients [4].

The concept of ultrastaging implies that lymph nodes be systematically analysed using serial sectioning and IHC. However, histological and/or molecular techniques used to assess ultrastaging on all nodes are time consuming and expensive thus limiting its routine use. Hence, the concept of ultrastaging is inseparable from that of SLN biopsy [5]. In melanoma, breast cancer, vulvar and colon cancers, the relevance of SLN biopsy has been validated and is considered an alternative to comprehensive lymphadenectomy to assess lymph node status. Although accumulating data on SLN in uterine cancers are available, its validation remains a matter of debate especially for endometrial cancers due to the absence of consensus on the SLN technique. Moreover, few data are available on ultrastaging in uterine cancers. Therefore, the objective of the present review is to evaluate the contribution of ultrastaging in uterine cancers and its potential therapeutic implications.

### Concept of ultrastaging in uterine cancers

Despite favourable prognostic features, pelvic recurrence occurs in up to 15% of patients with early stage cervical cancer and histologically negative pelvic lymph nodes by routine examination using H&E staining [6,7]. Holmgren et al. suggested that some of these recurrences could be due to metastases not detected by routine H&E histology of lymph nodes, so-called "dormant" or "occult" metastases [8]. Hafner et al. reported that using routine H&E histology, the chances of identifying a tumour cell cluster of less than 3 cell diameters was only 1% [9].

In 2003, Dargent and Enria evoked the concept of micrometastases without clear histological definition in cervical cancer. They reported that the use of serial sectioning and IHC gave a possible tenfold increase in detecting micrometastases [10]. Moreover, as micrometastases were found when the technique was retrospectively applied to lymph nodes from women with recurrence, the relevance of both serial sectioning and IHC and its prognostic value emerged along with the concept of ultramicroscopic staging. In contrast to melanoma and breast cancer, there is an absence of universal agreement on the definition of lymph node metastases in cervical cancer. Following the Philadelphia Consensus Conference on sentinel nodes in breast cancer [11], definitions have been proposed: macrometastases as a single focus of metastatic disease per node measuring more than 2 mm, micrometastases as a focus of metastatic disease ranging from 0.2 mm to no more than 2 mm and, in accordance with Marchiolé et al, submicrometastases as metastases measuring no more than 0.2 mm (including the presence of a single non-cohesive tumor cell) [12]. SLN and pelvic lymph nodes are considered positive when they contain macrometastases, micrometastases or submicrometastases.

In 2004, histological validation of the concept of SLN biopsy in cervical cancer was demonstrated by Barranger et al [13]. Despite the small sample size, the contribution of serial sectioning and IHC to ultrastaging was evoked. In 2007, the same team validated the histological concept of SLN biopsy for endometrial cancer [14]. But, in contrast to cervical cancer, no standardization of the SLN procedure in endometrial cancer existed.

### Concerns on ultrastaging protocols

Ultrastaging protocols vary from one study to another and there is no validation of a standardized routine protocol to date. This has been emphasized recently in an editorial by Gien & Covens on quality control in sentinel node biopsy [15]. Results of ultrastaging depend on several factors including the technique of intraoperative histology, the technique of serial sectioning and the antibodies used for IHC.

Imprint cytology has been proved to have a low accuracy to detect micrometastases in both cervical and endometrial cancer but has the advantage of preserving tissue for definitive histology [16]. However, no trial has compared the accuracy of imprint cytology to that of frozen section. So far, insufficient data are available to evaluate the contribution of molecular techniques to assess metastases intraoperatively. Yet, detecting metastases during surgery is required to extend lymphadenectomy to the paraaortic area.

Serial sectioning is often mentioned in the material and methods section of published reports but the exact histological technique is rarely described. Under the term of serial sectioning various conditions exist. The number of levels ranged from one additional level to up to five additional levels and the interval between levels ranged from 40 to 250 μm [17]. However, the technique of serial sectioning is crucial for adequate staging and reducing the false negative rate [1,14]. Even though most of the publications on SLN series report using cytokeratin (CK) antibodies for IHC staining, serial sectioning with H&E staining is not systematically used [17]. In endometrial cancer some studies have confirmed that the number of histological sections plays a crucial role in detecting metastases. Reich et al using serial-step section at 600 to 800 μm intervals, reported that metastases measuring less than 2 mm were detected in up to 50% of patients [2].

Most studies purport that the optimal method for ultrastaging includes an IHC. The signal amplification produced by immunodetection facilitates disease detection compared with H&E. In uterine cancers, the types of antibodies used for IHC staining varied according to the series. Although the majority of authors used anti-CK AE1 and AE3, some authors recommended anti-pancytokeratine KL1. In contrast, CAM antibodies are rarely used even though this antibody differentiates true metastases from mesothelial staining.

In cervical cancer, Lentz et al [18] using the IHC without serial sectioning reported that IHC detected micrometastases in 19 out of a series of 132 women with 3,106 negative lymph nodes on routine histology (15%, 95% interval confidence (IC): 9%-22%). Silva et al emphasized the contribution of IHC in detecting micrometastases in a series of 52 patients with stage I-II cervical cancer [19]. In their study, IHC detected micrometastases in five out of 98 negative SLN. Barranger et al in the report on histological validation of SLN in cervical cancer noted that micrometastases were found in two of the five patients with metastases with the use of IHC [13]. As underlined by Euscher et al, the ultrastaging protocol for negative sentinel node on routine histology consisted of 3 consecutive sections (5 μm thick), each obtained at 5 levels (40 μm interval). Then, a first section of each level was stained with H&E. The two unstained sections at each level were available for additional analysis when atypical cells were detected on H&E. When the five additional H&E stained levels were negative, then an unstained section from the first level was stained with a keratin cocktail to confirm the negative histologic impression. This keratin cocktail was composed of 4 antibodies: AE1/AE3, CAM 5.2, Cytokeratin MNF116, Keratin 8 and 18 allowing both to detect metastasis as well as to differentiate true metastasis from benign inclusion [17]. In breast cancer, Cote et al., evaluated the contribution of serial sectioning (2 sections from each of six levels) and immunohistochemistry (2 anticytokeratins AE-1 and a CAM 5.2) to the routine histology (ref) and detected 20% of additional micrometastasis [1].

In a case control study in women with cervical cancer, Marchiole et al showed that IHC detected micrometastases in 23% of patients [12]. These authors also underlined the risk of false positive cases of micrometastases related to benign glandular inclusions. Marchiolé et al. noted that even RT-PCR had a better sensitivity than IHC, this is counter balanced by a lack in specificity. Indeed, it is not possible to differentiate macrometastasis from benign glandular inclusion using only RT-PCR. Moreover, even a correlation has been established between the number of copy cells and the size of metastases, RT-PCR is enable to differentiate true macrometastases from multiple micrometastasis or submicrometastasis.

Similar results have been observed by Nickles-Fader et al [20] reporting that one of the three suspected micrometastases corresponded to mesothelial staining.

In endometrial cancer, similar results showing that IHC based on CK staining may improve the sensitivity of detecting metastasis compared with H&E staining have been reported [21,22]. In a pilot study using H&E histology and IHC without serial sectioning [21], 12.5% of patients with negative pelvic lymph nodes on H&E exhibited metastases by IHC. Niikura et al [23] using serial sectioning and IHC noted that micrometastases or isolated tumour cells were detected in four out of 24 negative SLN (5% of patients) and in four out of 1,350 non SLN. These results have been confirmed by other teams, Fersis et al. [24] and Pelosi et al. [25]. Finally, Barranger et al in their report on histological validation of SLN in endometrial cancer, showed that IHC and serial sectioning detected micrometastases in three out of five patients with lymph node metastases [13].

Advances in the understanding of cellular biology combined with developments in molecular technology have provided new methods for the detection of metastatic cancer cells, which are likely to be more sensitive than conventional histology. This molecular biology-based ultrastaging of cancer is already part of the standard management of patients with hematologic malignancies. However, the search for minimal residual disease by means of molecular biology techniques in solid tumours remains controversial. In melanoma, although ten studies have been performed and thousands of patients enrolled, there is no consensus on whether molecular biology-based detection of micrometastases has a prognostic power reliable enough to be implemented in routine clinical practice [26]. In a 2001 study on cervical cancer, Van Trappen et al evaluated the use of RT-PCR to detect CK-19 in pelvic lymph nodes [27]. CK-19 expression was correlated to lymph node status. However, Coutant et al reported a low correlation between CK-19 expression by RT-PCR and SLN status [16]. Recently, Yuan et al [28] using the same technique as Van Trappen et al reported a wide overlapping in CK-19 expression between positive and both negative SLN and non-SLN. Yuan et al suggested that detection by RT-PCR of squamous cell carcinoma antigen (SCCA) was more accurately associated with lymph node status than CK-19 expression. The expression levels of squamous cell carcinoma antigen (SCCA), CK 19 and glyceraldehyde-3-phosphate dehydrogenase (GAPDH) mRNA in 178 samples were assessed by PCR [28]. The authors used a fully quantitative real-time RT-PCR and avoid amplification and detection of CK 19 genes [28]. Their results showed that CK19 is not a suitable marker for molecular diagnosis of SLN metastasis in cervical cancer because of its low specificity in normal lymph node and showed that SCCA is a better marker than CK19 [28].

Hafner et al [9] suggested that E6 expression was linked to lymph node status but, as in previous studies [27,28], there was a high overlapping of values between positive and negative lymph nodes. Coutant et al reported that HPV DNA screening in SLN by means of PCR might help to identify patients at risk of lymph node metastases and recurrence although HPV DNA was noted in only 46.7% of positive SLN and in 13.6% of negative SLN [29]. While molecular techniques (such as RT-PCR) may be more sensitive than IHC, they carry a high false positive rate [30]. Indeed, Van Trappen et al underlined that specific tumour DNA found in histologically normal lymph nodes may originate from dead cell material or macrophages and that viral DNA can be found in various cell types thus limiting its usefulness as a molecular marker for micrometastases [27]. Marchiolé et al noted that even RT-PCR had a better sensitivity than IHC though this is counterbalanced by a lack of specificity [12]. Moreover, it is not possible to differentiate macrometastasis from benign glandular inclusion using only RT-PCR. In addition, even if a correlation has been established between the number of copy cells and the size of metastases, RT-PCR lacks accuracy in differentiating true macrometastases with proved prognostic value from multiple micrometastases or submicrometastases with questionable clinical relevance.

In endometrial cancer few data are available on the contribution of molecular techniques to detect lymph node metastases. Fishman et al were the first to report a high CK-20 expression by RT-PCR in primary tumours and in pelvic lymph nodes. Among the 18 patients with negative pelvic lymph nodes by routine H&E histology, six (33%) were CK-20 positive suggesting a potential contribution of molecular biology in assessing lymph node status. So far, no data are available on CK-20 expression by RT-PCR in SLN in patients with endometrial cancer [31].

### Incidence of micrometastases and potential clinical implications in patients with uterine cancers

The definition of micrometastases is rarely clearly mentioned in published reports representing a potential bias in the interpretation of their prognostic relevance. Moreover, as previously noted, the incidence of micrometastases can differ significantly according to the histological and biological technique used.

In cervical cancer, whatever the histological technique used for detecting lymph node involvement, the rate of macrometastases varied from 7.1% to 42% (table 1, 2).

**Table 1:** Ultrastaging of sentinel lymph node using H&E and IHC in patients with cervical cancer

Study	Year	Method of analysis	Nb of patients	FIGO stage	Macrometastatic SLN (%)	Micrometastatic SLN (%)
**Lambaudie**	2003	H&E +IHC	12	IA2-IB1	2 (18.2)	0
**Niikura**	2004	H&E +IHC	20	IB1-IIA	2 (10)	0
**Martinez Palones**	2004	H&E +IHC	23	IA2-IIA	3 (13)	0
**Kraft**	2006	H&E +IHC	54	IB1-III	21 (42)	na

**Total**			109		28 (25.7)	

SLN: sentinel lymph node; H&E: hematein eosin staining; IHC: immunohistochemy; SS: serial sectioning; HPV: human papilloma virus; na: not available

**Table 2:** Ultrastaging of sentinel lymph node using H&E, serial sectioning and IHC in patients with cervical cancer

Study	Year	Method of analysis	Nb of patients	FIGO stage	Macrometastatic SLN (%)	Micrometastatic SLN (%)
**Lantzsch**	2001	H&E +SS+IHC	14	IB1	1 (7.1)	0
**Plante**	2003	H&E +SS+IHC	70	IA-IIA	8 (13.1)	3 (37.5)
**Dargent**	2003	H&E +SS+IHC	70	IA1-IIB	19 (30.2)	9 (47.4)
**Hubalewska**	2003	H&E +SS+IHC	37	I-IIA	5 (13.5)	na
**Pijpers**	2004	H&E +SS+IHC	34	early	12 (36.3)	4 (33)
**Silva**	2005	H&E +SS+IHC	56	IA2-IIA	17 (32.7)	3 (17.6)
**Rob**	2005	H&E +SS+IHC	183	IA2-IB2	35 (21.9)	na
**Angioli**	2005	H&E +SS+IHC	37	IB1	6 (23)	0
**Di Stefano**	2005	H&E +SS+IHC	50	IA2-IIA	9 (20)	2 (22.2)
**Frumovitz**	2006	H&E +SS+IHC	50	IA2-IB1	9 (18.8)	na
**Wang**	2006	H&E +SS+IHC+CK19PCR	46	early	18 (39)	7 (38.9)
**Yuan**	2007	H&E +SS+IHC	81	IB1-IIA	17(20.9)	4 (23.5)
**Coutant**	2007	H&E +SS+IHC+HPV DNA	59	IA-II	15 (25.4)	3 (20)
**Lee**	2007	H&E +HPV DNA	57	IB-IIA	11 (19.3)	na
**Hauspy**	2007	H&E +SS+IHC	39	IA1-IIA	2 (5.2)	na
**Bats**	2007	H&E +SS+IHC	25	IA2-IA1	3 (12)	1 (33)

**Total**			908		187 (20.6)	36 (19.2)

SLN: sentinel lymph node; H&E: hematein eosin staining; IHC: immunohistochemy; SS: serial sectioning; HPV: human papilloma virus; na: not available

Four studies have performed a histological analysis of lymph nodes using H&E and IHC [32-35]. In the series of Kraft et al including 54 patients, overall rate of macrometastases was 42% but there was no mention of the rate of micrometastases [35]. In the three remaining studies including 65 patients, the rate of macrometastases varied from 10% to 18.2% but none of the studies reported detecting micrometastases. Although the total number of patients included in these series was low, it is possible to suggest that H&E and IHC are insufficient to detect micrometastases.

Thirteen studies have used the combination of H&E, serial sectioning and IHC [10,19,28,36-44]. In four of the thirteen studies no attempt to evaluate the presence of micrometastases was noted. In the remaining nine studies involving 356 patients the rate of macrometastases varied between 7.1% and 36.3% with a mean value of 25.8% (92/356). Among patients with lymph node metastases, the percentage of women with micrometastases ranged from 0% and 47.4% with a mean value of 28.3%. Therefore, at least one quarter of patients with lymph node metastases exhibited micrometastases.

Few data are available on the contribution of molecular biology to detect micrometastases. In Wang et al's series, the combination of H&E, serial sectioning, IHC and CK-19 expression by RT-PCR detected macrometastases in 18 out of 46 patients (39%) with lymph node metastases and micrometastases in 7 out of the 18 patients (38.9%) with macrosmetastases [45]. For Coutant et al, HPV DNA analysis in conjuction with H&E, serial sectioning and IHC detected macrometastases in 15 out of 59 patients including three with micrometastases (20%) [29].

In endometrial cancer, whatever the histological technique used for detecting lymph node involvement, the rate of macrometastases varied from 0% to 22% (table 3, 4).

**Table 3:** Ultrastaging of sentinel lymph node using H&E or H&E and IHC in patients with endometrial cancer

Study	Year	Method of analysis	Nb of patients	FIGO stage	Macrometastatic SLN (%)	Micrometastatic SLN (%)
**Burke**	1996	H&E	15	I-II	2 (13)	na
**Echt**	1999	H&E	8	I-IV	na	na
**Holub**	2004	H&E	25	I	2 (8)	na
**Raspagliesi**	2004	H&E	18	I-III	4 (22)	na
**Altgassen**	2007	H&E	25	I-II	2 (8)	na
**Frumovitz**	2007	H&E	18	I-II-III	0	na
**Li**	2007	H&E	20	I-II-III	2 (10)	na
**Pelosi**	2003	H&E+IHC	16	I	3 18)	3 (18)
**Niikura**	2006	H&E+IHC	20	I-II-III	4 (20)	4 (20)

H&E: hematein eosin staining; IHC: immunohistochemy; SLN: sentinel lymph node; na: not available

**Table 4:** Ultrastaging of sentinel lymph node using H&E, serial sectioning and IHC in patients with endometrial cancer

Study	Year	Method of analysis	Nb of patients	FIGO stage	Macrometastatic SLN (%)	Micrometastatic SLN (%)
**Maccauro**	2005	H&E+SS+IHC	26	I-III	4 (15)	0
**Delpech**	2007	H&E+SS+IHC	23	I-II	5 (21)	3 (13)
**Delaloye**	2007	H&E+SS+IHC	60	I-II-III	8 (13)	0
**Lopes**	2007	H&E+SS+IHC	40	I-II	5 (12)	2 (5)
**Ballester**	2008	H&E+SS+IHC	46	I-II-III	3 (6)	7 (15)
**Bats**	2008	H&E+SS+IHC	43	I-II-III	8 (18)	2 (4)

H&E: hematein eosin staining; SS: serial sectioning; IHC: immunohistochemy; SLN: sentinel lymph node; na: not available

Seven studies reported a histological analysis of lymph nodes using H&E [46-52]. The rate of macrometastases varied from 8% to 22% but none of the studies reported the detection of micrometastases. As for cervical cancer, the use of H&E alone was unable to detect micrometastases confirming that this technique is insufficient to stage endometrial cancer. The combination of H&E to IHC was used in two studies [23,25]. The contribution of IHC was particularly relevant as respectively 18% and 20% of patients were upstaged after detection of micrometastases.

Six studies have used the combination of H&E, serial sectioning and IHC to detect micrometastases [14,53-57]. The rate of micrometastases varied from 0% to 15%. Among the 238 patients with endometrial cancer, the overall rate of lymph node metastases was 19.7% including 5.8% with micrometastases. The most striking data was observed in the series of Ballester et al showing that 10 out of the 46 patients with endometrial cancer exhibited lymph node metastases [56]. In their study, three of the ten metastases corresponded to macrometastases and seven to micro- or submicrometastases. All the three cases of macrometastases and the three additional micrometastases were detected by H&E while three micrometastases and one submicrometastases were diagnosed by serial sectioning and IHC.

The relation between micrometastases and the risk of recurrence and prognosis have been demonstrated in an increasing number of malignancies including breast cancer [1,58-60], vulvar cancer [61-63]; gastric cancer [64], oesophageal cancer [65], colon cancer [66,67], prostate cancer [68], and melanoma [26] suggesting that micrometastases should be an indication for adjuvant therapy.

In cervical cancer, although the prognostic relevance of micrometastases has not yet been established, Juretzka et al recommend adjuvant radiotherapy in the event of detection of micrometastases [69]. Marchiolè et al found that the relative risk of recurrence in presence of true micrometastases (focus of metastatic disease ranging from 0.2 mm to no more than 2 mm) was 2.30 (CI: 1.65-3.20, p < 0.01) and 2.22 (CI: 1.30-3.80, p = 0.09) in the presence of submicrometastases (focus of metastatic disease no more than 0.2 mm including the presence of single non cohesive tumour cells) [13]. These authors addressed the issue of adjuvant therapy in patients with both lymphovascular space involvement and micrometastases [13]. However, despite a high incidence of micrometastases in cervical cancer, Coutant et al failed to demonstrate a relation between the presence of micrometastases or submicrometastases and the recurrence rate, probably due to the small sample size and a relative short follow-up [29].

In early stage endometrial cancer, Yabushita et al. [22] analyzed the relation between disease recurrence and presence of micrometastases by IHC in pelvic lymph nodes. Although in their report, the term micrometastases is used to refer to metastases in which tumor cells were detected only by the IHC method and the term occult metastasis refers to the presence of tumor cell fragments, the authors found that micrometastases in lymph node was associated with recurrence of disease in univariate (p < 0.0001) and multivariate analysis (p = 0.009). However, as for cervical cancer, the debate on whether the detection of micrometastases could be an indicator of adjuvant therapy continues.

## Conclusion

Although accumulating data emphasize the contribution of serial sectioning and IHC to detect micrometastases, the clinical implications of ultrastaging on adjuvant therapy remains a matter of debate in uterine cancers.

## Competing interests

The authors declare that they have no competing interests.

## Authors' contributions

CB,  the first author collected and analyzed all the data and wrote the article. CC corrected and supervised the article. MB collected local data. J-GF collected local data. RR supervised the statistical analysis. SU and ED supervised this work and corrected the article. All authors read and approved the final manuscript.
